# Radon transfer from thermal water to human organs in radon therapy: exhalation measurements and model simulations

**DOI:** 10.1007/s00411-019-00807-z

**Published:** 2019-06-29

**Authors:** W. Hofmann, R. Winkler-Heil, H. Lettner, A. Hubmer, M. Gaisberger

**Affiliations:** 1 Biological Physics, Department of Chemistry and Physics of Materials, University of Salzburg, Hellbrunner Str. 34, 5020 Salzburg, Austria; 2 Institute of Physiology and Pathophysiology, Paracelsus Medical University, Strubergasse 21, 5020 Salzburg, Austria; 3 Gastein Research Institute, Paracelsus Medical University, Strubergasse 21, 5020 Salzburg, Austria; 4 Ludwig Boltzmann Institute for Arthritis and Rehabilitation, Strubergasse 21, 5020 Salzburg, Austria

**Keywords:** Radon therapy, Thermal water, Radon skin transfer, Exhalation measurements, Biokinetic simulations

## Abstract

The transfer of radon from thermal water via the skin to different human organs in radon therapy can experimentally be determined by measuring the radon activity concentration in the exhaled air. In this study, six volunteers were exposed to radon-rich thermal water in a bathtub, comprising eleven measurements. Exhaled activity concentrations were measured intermittently during the 20 min bathing and 20 min resting phases. Upon entering the bathtub, the radon activity concentration in the exhaled breath increased almost linearly with time, reaching its maximum value at the end of the exposure, and then decreased exponentially with time in the subsequent resting phase. Although for all individuals the time-dependence of exhaled radon activity was similar during bathing and resting, significant inter-subject variations could be observed, which may be attributed to individual respiratory parameters and body characteristics. The simulation of the transport of radon through the skin, its distribution among the organs, and the subsequent exhalation via the lungs were based on the biokinetic model of Leggett and co-workers, extended by a skin and a subcutaneous fat compartment. The coupled linear differential equations describing the radon activity concentrations in different organs as a function of time were solved numerically with the program package Mathcad. An agreement between model simulations and experimental results could only be achieved by expressing the skin permeability coefficient and the arterial blood flow rates as a function of the water temperature and the swelling of the skin.

## Introduction

In the Gastein valley, Austria, natural radon-rich water in springs and radon vapor in the thermal gallery have been successfully used in the past for the treatment of various rheumatic diseases, most notably ankylosing spondylitis (Shehata et al. 2006; van Tubergen et al. 2001). Although the physiological and cellular mechanisms triggered by very low radiation doses resulting from the radon exposures are still not yet fully understood, several studies have proved the effectiveness of the radon treatment (Bernatzky et al. 1994; Falkenbach 2000; Falkenbach et al. 2005; Franke et al. 2000, 2007; Mitsunobu et al. 2003; Moder et al. 2011). Three different main therapeutic regimes are presently used: (1) exposure to radon in a thermal bath, (2) exposure to radon and its progeny in the thermal gallery (“Gasteiner Heilstollen”), a former gold and silver mine, and (3) exposure to radon vapor in a specially designed exposure chamber (“vapor bath”) (note: inhalation of radon progeny in thermal and vapor baths play only a minor role).

(1) In the thermal bath, patients are sitting in a bathtub (37–40 °C) for a period of 20 min (on average ten treatments) with a radon activity concentration in water of about 900 kBq m^−3^. (2) In the thermal gallery, patients are exposed to radon and radon progeny for 60 min (on average ten treatments) at an average radon activity concentration in air of about 45 kBq m^−3^ (37–41.5 °C and 70–99% RH). (3) In the vapor bath, patients are exposed to radon vapor (37 °C) in a small exposure chamber for a period of 20 min (on average ten treatments) with radon activity concentrations in air ranging from 30 to 300 kBq m^−3^ (average value: 90 kBq m^−3^). Here, the exposure conditions are similar to those in the thermal gallery, except that the head of the patient remains outside the exposure chamber, thereby significantly reducing the inhalation of radon and its progeny.

The therapeutic response in these radon treatment schemes has traditionally been attributed to the radiation doses received by specific organs and tissues of the human body although the detailed mechanisms of radiation action remained largely unclear. In the case of thermal water therapy, the radiological exposure pathways to human organs and tissues are the uptake of radon through the skin and its subsequent distribution throughout the human body via the bloodstream. In addition, inhalation of radon and its short-lived progeny in the treatment facilities may also slightly contribute to these organ doses. Recently, however, Tempfer et al. (2010) proposed an immune response caused by irradiation of the deeper layers of the skin by radon progeny adsorbed to the skin as an alternative explanation for the therapeutic effectiveness of the thermal water treatment.

Medical interpretation of any therapeutic mechanisms triggered by radon exposure requires a thorough analysis of the radiation doses incurred by sensitive organs or tissues of the human body. Current lung dosimetry models provide reliable tools for dose assessment via radon and radon progeny inhalation (Hofmann and Winkler-Heil 2011, 2015; James et al. 2004; Marsh and Birchall 2000; Winkler-Heil et al. 2007), and radiation doses due to radon progeny deposited on skin surfaces were presented by Tempfer et al. (2005, 2010). While various organ dose models for incorporated radon have been published in the past (Hofmann et al. 1990; Kendall and Smith 2002; Khursheed 2000; Leggett et al. 2013; Peterman and Perkins 1988; Sakoda et al. 2010), these models are restricted to radon inhalation and exhalation via the lungs. In a previous modeling attempt, the Peterman and Perkins (1988) model had been extended to incorporate the water-skin-blood transfer of radon for the simulation of the radon activity concentrations in exhaled breath and in various organs and tissues of the human body (Hofmann and Winkler-Heil 2010). More recently, Sakoda et al. (2016) evaluated the uptake of radon through the skin from thermal water by incorporating an additional skin compartment to the biokinetic model for noble gases proposed by Leggett et al. (2013) specifically for radon inhalation.

Radon transfer through the skin and its subsequent distribution among the organs and tissues of the human body can experimentally be determined only by indirect methods, i.e. by measurement of radon exhalation via the lungs or radon excretion in urine, or by measurements of the radon activity concentration in venous blood. Regarding urine excretion, Kavasi et al. (2011) concluded that although radon was detected in urine after thermal water treatment, they could neither reject nor confirm the hypothesis of radon absorption through the skin. While Grunewald et al. (1999) reported blood activity concentrations after radon exposure in thermal water comparable to those in the exhaled air, previous measurements of the radon activity concentration in blood revealed that radon levels in the Gastein thermal baths were too low to allow any statistically significant conclusion (Hofmann et al. 1999). Thus, the only remaining experimental approach to determine any radon transfer through the skin appeared the measurement of radon in the exhaled breath.

Radon exhalation measurements during thermal water treatment in radon spas have been reported by Grunewald and Grunewald (1995), Grunewald et al. (1999, 2009), Hofmann et al. (1999), Hofmann and Winkler-Heil (2010), and Tempfer et al. (2005). The primary goal of the study described in Grunewald et al. (2009) was to measure the “radon transfer”, defined as the radon activity taken up by the human body via the skin and exhaled via the lungs, considering the human body by a black box. On the other hand, Hofmann and Winkler-Heil (2010) used the measured radon exhalation curves as a function of time during bathing and resting, to determine the transfer rates water-to-skin and skin-to-blood and to calculate radon activity concentrations in different organs and tissues.

In this previous attempt (Hofmann and Winkler-Heil 2010), the multi-compartment model RADMOD was used to simulate incorporation, distribution and clearance of radon in radon therapy patients. In this model, based on the biokinetic model of Peterman and Perkins (1988), lungs and skin represented the incorporation compartments, lungs, skin and bladder the clearance compartments, blood the distribution compartment, and 12 different organs and tissues the target compartments (Hofmann and Winkler-Heil 2010). Model predictions of the exhaled radon activity concentration agreed quite well with the experimental observations. While the radon activity concentration in the exhaled breath reached a saturation value towards the end of the exposure, it dropped significantly in an exponential fashion with time within a few minutes during the subsequent resting phase.

Since the measured data used in that study were obtained in a single volunteer, this database was insufficient to be used for model validation. Hence the objective of the present study was to repeat these measurements with a larger number of participants. In addition, the model by Peterman and Perkins (1988) was replaced by that of Leggett et al. (2013), because the latter model uses the most recent data on transfer coefficients, blood flow rates and organ masses and volumes.

Besides the radon transfer through the skin to the blood, either from water or air, another incorporation pathway of radon is inhalation. Because of the negligible radon activity concentration in the inhaled ambient air, the measurement of radon exhalation of individuals in the thermal water bath of the present study allowed determination of the radon transfer rate from radon in water via the skin to the blood without any interference from the inhalation pathway.

Thus, the objectives of the present study were (1) to measure the exhaled radon activity concentration as a function of time during and after radon exposure of several volunteers in a thermal bath, (2) to extend the biokinetic radon distribution model of Leggett et al. (2013) to radon uptake through the skin, (3) to calculate radon activity concentrations in several organs of the human body during and after radon exposure, and (4) to determine the experimentally observable radon transfer rate from the skin to the exhaled air.

## Materials and methods

### Radon exhalation measurements

The experimental methods used for the radon exhalation measurements of the present study have already been described in the companion paper of Lettner et al. (2017), and thus only those features of the experimental methods important for the understanding of the measured exhalation curves are discussed below.

While sitting in the thermal bath (volume: 600 L, temperature: between 37 and 40 °C), the test person breathes through a breathing mask with an inlet and an outlet tube, permitting inhalation and exhalation through the mouth. The volunteers inhaled practically radon-free outside air (average radon activity concentration was approximately 10 Bq m^−3^) and exhaled radon from the lungs either into the room air (average radon activity concentration approximately 100 Bq m^−3^) or in pre-specified time intervals (about 4 min) into a radon-tight aluminum foil-synthetic bag collecting the radon-containing exhalation air for subsequent analysis. During inhalation, the outlet tube was closed by a valve, so that only outside air is inhaled. Likewise, the inlet tube was closed during exhalation, so that only air from the lungs is exhaled into the container. After one breath, which was sufficient to obtain measurable radon activity concentrations, the radon-containing plastic bag was disconnected from the breathing mask and, after a pre-specified time interval, replaced by a new one for the next sampling. This sampling protocol allowed the measurement of exhaled radon activity concentrations in approximately 4 min intervals during the 20 min bathing period. The exhalation sampling was then continued for another 20 min after the volunteer has left the thermal bath, to track the decrease of the radon activity concentration in the respiratory air. During the resting phase, the volunteers still inhaled practically radon-free outside air through the inlet tube.

The plastic containers with the radon were then analyzed in a laboratory in Salzburg, Austria, by alpha liquid scintillation counting with Lucas cells of 250 ml and a Pylon AB-5 radon monitor (Pylon Instruments), applying a pressure correction to account for the air pressure difference between the Gastein area (sampling) and Salzburg (measurement).

At the switch from inhalation to exhalation, the inlet tube was closed by a valve, leaving an instrumental dead space *V*_D_ between the two valves of 110 cm^3^ (there was an additional dead space of 40 cm^3^ between the valve of the outlet tube and the mouth), which had been filled with outdoor air during the preceding inhalation phase. Thus, at the onset of exhalation, the volunteers first exhaled air from this dead space, followed by the radon-containing air from the respiratory tract. As a result, the exhaled radon activity concentration was diluted by the dead space air. If the radon activity concentration of the outside air is negligibly small compared to the radon activity concentration in the respiratory tract, which was the case in the present study, the exhaled radon activity concentration will represent the fraction (*V*_T_–*V*_D_)/*V*_T_ of the exhaled tidal volume *V*_T_. Thus, the measured exhaled radon activity concentration has to be corrected for the contribution of the instrumental dead space (Lettner et al. 2017). Likewise, during inhalation, the inhaled outdoor air was mixed with the radon activity remaining in the dead space at the end of exhalation. However, this effect increased the radon activity in the respiratory air compartment only by a few percent, and thus had been neglected in the present analysis.

As a result of radon emanation from the water surface and, to a lesser extent, of radon transfer to the human body, the radon activity concentration in water was continuously reduced during the bathing phase by a few percent (Lettner et al. 2017). Hence, the radon activity concentrations of the thermal water in the bathtub during the exhalation measurements (Table 1) represent average values obtained from measurements at the beginning and at the end of the exposure phase.

**Table 1:** Average radon activity concentrations in thermal water, derived from two measurements at the beginning and at the end of bathing

Subject	Gender	Average radon activity concentration (MBq m^−3^)
1	F	0.720/0.880
2	F	0.830/0.860
3	F	0.960
4	F	0.870/0.840
5	M	0.890
6	M	0.870/0.700/0.815

Average radon activity concentrations were approximately 10 Bq m^−3^ in the inhaled outdoor air and approximately 100 Bq m^−3^ in the air of the bathroom

The individual body characteristics and respiratory parameters of the volunteers participating in this study are listed in Table 2. Altogether six volunteers participated in the present study. The participants were of either gender (four females and two males) with ages between 26 and 40 years. The age of the test subjects was restricted to a relatively narrow range to minimize the effect of age on radon uptake through the skin. All selected volunteers were healthy individuals who normally would not receive any radon therapy treatment. Altogether eleven exhalation measurements were carried out. Four volunteers were measured twice to investigate any individual temporal variations, two of them for an extended exposure period of 30 min to demonstrate the saturation effect of the exhalation curve. To check whether the water temperature affected the radon transfer, one test person spent 10 min in radon-free water at the same temperature prior to the radon exposure.

**Table 2:** Individual respiratory parameters and body characteristics of the six volunteers participating in this study

SN	Age (year)	Height (cm)	Mass (kg)	BSA (m^2^)	BF (kg)	FRC (l)	*V*_RT_^a^ (l)	*V*_T_ (l)	RF (min^−1^)	RMV (l h^−1^)
1	36	168	58	1.65	17.7	2.78	3.25	0.56	15.3	514
2	26	160	52	1.52	10.8	2.61	3.05	0.52	20.5	640
3	28	160	57	1.59	14.3	2.61	3.05	0.52	13.9	434
4	32	163	62	1.68	19.0	2.62	3.06	0.52	15.0	468
5	40	184	90	2.14	21.0	3.58	4.19	0.72	14.0	605
6	28	187	83	2.08	19.7	3.54	4.14	0.70	11.1	466

*SN* subject number, age, height, mass, *BSA* body surface area, *BF* body fat, *FRC* functional residual capacity, *V*_*RT*_ respiratory tract volume, *V*_*ET*_ extrathoracic volume, *V*_*T*_ tidal volume, *RF* respiratory frequency, *RMV* respiratory minute volume

^a^*V*_RT_ = FRC + *V*_ET_

While breathing frequencies were directly measured by spirometry methods, corresponding tidal volumes *V*_T_ were derived by integration over the measured airflow curves and from measured forced vital capacity (FVC) values by assuming a constant empirical ratio between *V*_T_ and FVC (ICRP 1994). The functional residual capacities (FRC) for each volunteer were obtained from empirical relationships between FRC and age and height for females and males (ICRP 1994). The derived data were consistent with the assumption of a constant relationship between FRC and FVC (ICRP 1994). Since radon transport through the skin depends on body surface area and fat content of the skin, the results of body surface area calculations and body fat content measurements are also listed in Table 2. The body surface area of the volunteers was calculated with the method of Mosteller (1987) while the body fat was measured by bioimpedance analysis with a Nutriguard S device.

The main sources of the uncertainties of the radioactivity measurements of the exhaled air were the statistical errors of the radon activity concentrations and the random variations of the breathing parameters of the volunteers during a given exposure session. These combined errors were estimated to range from about ± 5 to 15%.

### Radon transfer and organ distribution model

The transfer and organ distribution model used in the present study to describe the uptake of radon via the skin, its transfer to blood, its subsequent distribution among human organs and tissue via the bloodstream, and its final exhalation, is based on the biokinetic model for noble gases of Leggett et al. (2013), with specific application to radon. In this model, human organs and tissues are represented by eight compartments, three of them, i.e. fat, bone and breast, are further divided into two sub-compartments, which are connected with the arterial (Blood-A) and venous (Blood-V) blood compartments. Although the Peterman and Perkins (1988) model consists of only one blood compartment, simulations of the radon exhalation rates following continuous exposure to a high radon activity concentration for 8.5 h with both models gave nearly identical results (Leggett et al. 2013).

In the Leggett et al. (2013) model, radon entering the respiratory tract (RT) during inhalation is assumed to rapidly reach equilibrium between the RT air and pulmonary blood, with relative concentrations determined by the blood-to-air partition coefficient. Radon retained in the pulmonary blood after inhalation is then distributed in arterial blood to organs and tissues in proportion to the percentage of the cardiac blood supply to each tissue. The radon transfer coefficients from organs or tissues to venous blood are determined by the blood perfusion rates, the volume of the organs, and the tissue-to-blood partition coefficients. Radon is then carried in the venous blood to the pulmonary blood, and finally, due to the exchange between pulmonary blood and RT-air, is exhaled. Because of the assumption of equilibrium between RT-air and pulmonary blood, the arterial and venous blood compartments represent the non-pulmonary blood pool.

The radon activity in a given organ or tissue, resulting from the transfer from arterial blood, absorption in that organ or tissue, and transfer to venous blood can be described by a differential mass balance equation. The dependence of the radon activity *Q*_*i*_ in compartment *i* is given by:

1 $$ {\text{d}}Q_{i} / {\text{d}}t = \left( {F_{i} /V_{\text{BA}} } \right) \, Q_{\text{BA}} - \left( {F_{i} /P_{i} V_{i} } \right) \, Q_{i} - \lambda_{\text{r}} Q_{i} $$

where *F*_*i*_ is the blood flow rate related to the cardiac output, *V*_BA_ the volume of arterial blood, *Q*_BA_ the radon activity in arterial blood, *P*_*i*_ the tissue-to-blood partition coefficient, *V*_*i*_ the organ volume, and *λ*_r_ the radioactive decay constant. Thus, the transfer coefficient from arterial blood to tissue is *F*_*i*_/*V*_BA_ and the transfer coefficient from tissue to venous blood is *F*_*i*_/(*P*_*i*_*V*_*i*_). Due to a water temperature between 37 and 40 °C in the thermal bath and the resulting dilatation of the blood vessels, blood flow rates through the different organs will be higher in the present experimental setting than proposed by Leggett et al. (2013) for normal air temperatures.

Based on the assumption of equilibrium between Blood-A and RT-air, the change of the radon activity in RT-air, *Q*_RT_, is given by:

2 $$ \begin{aligned} {\text{d}}Q_{\text{RT}} / {\text{d}}t &= \lambda_{\text{E}} C_{\text{E}} V_{\text{RT}} - \lambda_{\text{E}} Q_{\text{RT}} + \left( {F/V_{\text{BV}} } \right)Q_{\text{BV}}\\&\quad - \left( {F\;P_{\text{BA}} /V_{\text{RT}} } \right)Q_{\text{RT}} - \lambda_{\text{r}} Q_{\text{RT}} \end{aligned} $$

where *λ*_E_ is the transfer coefficient from RT-air to the environment and vice versa, *C*_E_ the radon activity concentration in the environment, *V*_RT_ the volume of the respiratory tract, *F* the cardiac output, *V*_BV_ the volume of the venous blood, and *P*_BA_ the blood-to-air partition coefficient. The transfer coefficient from venous blood to RT-air is *F*/*V*_BV_ and the transfer coefficient from RT-air to arterial blood is *F P*_BA_/*V*_RT_).

The exhaled radon activity concentration *C*_EX_ in a single breath, which has been experimentally determined in the present study, is then given as the fraction of the radon activity concentration in the respiratory tract, *Q*_RT_*V*_T_/*V*_RT_, exhaled by tidal volume *V*_T_:

3 $$ C_{\text{EX}} = Q_{\text{RT}} /V_{\text{RT}} $$

After each exhalation, the radon activity in the respiratory air is slightly decreased by the exhaled activity, thus reducing also the amount of activity transferred to the arterial blood.

Since the Leggett et al. (2013) model considers only radon inhalation and ingestion but does not consider radon transfer through the skin, Sakoda et al. (2016) extended this model by an additional skin compartment, which transports radon from the water to the skin and further to the venous blood compartment. For comparison, in the earlier modeling study of Hofmann and Winkler-Heil (2010) radon was, based on the Peterman and Perkins (1988) model, directly transferred to the subcutaneous fat (fat 1) compartment without invoking a separate skin compartment. In the model developed in the present study the skin compartment was, therefore, combined with the subcutaneous fat compartment in a composite skin model.

Since the skin consists of the epidermis, the dermis with very little blood supply and which is low in fat, and the adipose hypodermis or subcutaneous tissue, which is rich in blood vessels and fat, the skin compartment was subdivided into a dermal skin (DS) compartment and a subcutaneous skin (SS) compartment. Because of its much higher blood flow and fat content, the radon transfer from water to blood in the skin takes place primarily in the adipose subcutaneous tissue layer. The diffusional transport of radon from water to the dermal skin compartment is characterized by a permeability coefficient and the surface area of the skin (Sakoda et al. 2016), with the stratum corneum as the rate-limiting outermost layer for the permeability (Potts and Francoeur 1990). The transfer rate from the DS to the SS compartment was adopted from the earlier modeling effort which was based on the Peterman and Perkins (1988) model (Hofmann and Winkler-Heil 2010). Thus, the pathway of radon from thermal water to venous blood is water–dermal skin–subcutaneous skin–venous blood.

The Leggett et al. (2013) model contains two fat compartments, labeled fat 1 and fat 2, with equal volume but different blood perfusion rates, to allow for a description of the two phases of long-term retention observed in humans, without, however, attributing each fat compartment to a specific organ or tissue. For comparison, the Peterman and Perkins (1988) model contains also two fat compartments with equal volumes and blood flow rates, where fat 1 with the lower transfer coefficient refers to subcutaneous fat. To preserve the compartmental structure of the Leggett et al. (2013) model, the fat 2 compartment in the Leggett et al. (2013) model, i.e. the fat compartment with the smaller transfer coefficient, was identified as the SS compartment in the present model (note: the choice of fat 1 instead of fat 2 does not appreciably affect the simulation results).

The dependence of the radon activity in the DS compartment, *Q*_DS_, as a function of exposure time *t* is then given by:

4 $$ \begin{aligned} {\text{d}}Q_{\text{DS}} / {\text{d}}t & = F_{\text{DS}} Q_{\text{BA}} /V_{\text{BA}} - F_{\text{DS}} Q_{\text{DS}} /\left( {P_{\text{DSB}} V_{\text{DS}} } \right) + K \, A_{\text{DS}} C_{\text{W}} \\ & \quad- K \,  A_{\text{DS}} Q_{\text{DS}} /\left( {P_{\text{SW}} V_{\text{DS}} } \right)  + \,k_{\text{DS}} Q_{\text{SS}} - k_{\text{DS}} Q_{\text{DS}} - \lambda_{\text{r}} Q_{\text{DS}} \\ \end{aligned} $$

where *F*_DS_ is the blood flow rate through the DS, *P*_DSB_ the DS-blood partition coefficient of radon, *V*_DS_ the volume of DS, *K* the permeability coefficient of radon in dermal skin, *A*_DS_ the surface area of the skin, *C*_W_ the radon activity concentration in water, *P*_SW_ the skin–water partition coefficient of radon, *k*_DS_ the transfer coefficient from DS to SS, and *Q*_SS_ the radon activity in SS. During the resting phase, the term *K A*_DS_*C*_W_ is set equal to zero.

Furthermore, the dependence of the radon activity in the SS compartment, *Q*_SS_, as a function of exposure time t is given by:

5 $$ \begin{aligned} {\text{d}}Q_{\text{SS}} / {\text{d}}t& = F_{\text{SS}} Q_{\text{BA}} /V_{\text{BA}} - F_{\text{SS}} Q_{\text{SS}} /\left( {P_{\text{SSB}} V_{\text{SS}} } \right) \\&\quad+ k_{\text{DS}} Q_{\text{DS}} - k_{\text{DS}} Q_{\text{SS}} - \lambda_{\text{r}} Q_{\text{SS}} \end{aligned} $$

where *F*_SS_ is the blood flow rate through the subcutaneous skin, *P*_SSB_ the SS-blood partition coefficient of radon, and *V*_SS_ the volume of SS.

In the systemic radon model of Leggett et al. (2013), different values for transfer coefficients, blood flow rates, and tissue and blood volumes and masses are listed for both male and female adults. Since such gender-specific differences could not be observed experimentally among the six volunteers of the present study (including four female and two male subjects), the parameter values for the adult females were consistently used for all simulations. Indeed, predicted exhaled radon activity concentrations are only slightly affected by these gender-specific differences. Since the volume of the respiratory tract of 3.858 l in the Leggett et al. (2013) model refers to male adults, it was scaled down to 3.131 l for female adults. Although one might also expect gender-specific differences for skin permeability and respiratory parameters not considered by the Leggett et al. (2013) model, experimental radon exhalation data do not allow the derivation of gender-specific differences due to apparent inter-subject variations observed in the present study.

Except for the two additional skin compartments in the present model, all other compartments were the same as in the models of Leggett et al. (2013) and Sakoda et al. (2016). As a result of our interpretation of the fat 2 compartment of the Leggett et al. (2013) model as the SS compartment, and the skin compartment of the Sakoda et al. (2016) model as the DS compartment, the majority of parameter values used in Eqs. 4 and 5 were taken from these models. For example, values of 0.4 and 11.0 were adopted for the skin-blood partition coefficients of radon in the DS and SS compartments, *P*_DSB_ and *P*_SSB_, respectively. Likewise, volumes of 1.84 l and 9.35 l were assumed for *V*_DS_ and *V*_SS_ and a value of 1.66 m^2^ for the total body skin surface area *A*_DS_. Furthermore, Sakoda et al. (2016) reported a value of 2.4 × 10^−7^ m s^−1^ for the skin permeability coefficient, *K*, representing the geometric mean of a lognormal distribution, derived from human volunteer exhalation data. Although this value refers specifically to the skin compartment in that model, it was also adopted as the default value for the present DS compartment. Finally, in the absence of any experimental data for the water-skin partition coefficient of radon, *P*_SW_, the skin-blood partition coefficient of 0.4 was also assumed for this parameter (note: no value was listed in Sakoda et al. 2016).

In the case of the transfer coefficient from DS to SS, *k*_DS_, and the arterial blood flow rates through DS, *F*_DS_, and SS, *F*_SS_, no parameter values were reported in the Leggett et al. (2013) and Sakoda et al. (2016) models as these pathways do not exist in both models.

The transfer coefficient *k*_DS_ from DS to SS in the Peterman and Perkins (1988) model refers to the transport of radon from the environment (air, water) to subcutaneous fat, and vice versa, and hence includes the transport through the DS compartment. Nevertheless, in the absence of any pertinent information, the original value of 0.18 h^−1^ (Peterman and Perkins 1988) was used as a default value in the present simulations.

In the present model, the total arterial blood flow rate to both skin compartments represents the sum of the blood flow rates through the fat 2 compartment of the Leggett et al. (2013) model and through the skin compartment of the Sakoda et al. (2016) model, i.e. 1.7% + 5.0% = 6.7%.

Assuming that the blood supply of the dermal skin is much smaller than that of the subcutaneous skin, say 10% versus 90%, yields blood flow rates of 0.7% and 6.0% of the cardiac output at room temperatures.

Furthermore, the above permeability coefficient refers to the diffusion of radon from water through a wet skin. In a dry skin, however, as in the case during the resting phase, diffusion of radon through the air-filled crevices of the dermal layer of the skin may be much higher. Since the diffusion coefficient in gases (air) is significantly higher than that in liquids, the permeability coefficient for the dry skin was increased to match the exponential decay of the exhalation curve during the resting phase. Likewise, this higher permeability coefficient was also applied to the radon diffusion from the non-exposed fraction of the skin to the ambient air during both exposure and resting phases.

While sitting in the bathtub, head and top of shoulders of the volunteers were not immersed in thermal water. Based on a surface area of the head of 7.5% of the human body (ICRP 2002) and assuming approximately 2.5% for the shoulders, the skin compartment was subdivided into an exposed skin compartment (90%) and a non-exposed skin compartment (10%) (Sakoda et al. 2016).

Thus, in the case of the non-exposed skin, Eqs. 4 and 5 still apply, except that *C*_W_ and *P*_SW_ in Eq. 4 are replaced by *C*_A_, the radon activity concentration in air, and *P*_SA_, the skin-air partition coefficient of radon, respectively. The distribution of the blood flows through the exposed and the non-exposed skin compartments was divided in proportion to their surface areas, i.e. 90–10%. The same procedure was applied to the determination of the volumes of the exposed and non-exposed skin compartments.

Since the exposure of the non-exposed DS compartment is negligibly small as compared to that of the exposed DS compartment (about 900 kBq m^−3^ in water vs. 100 Bq m^−3^ in air), the radon transfer to the non-exposed DS can safely be neglected. However, during the resting phase, the radon transfer from both exposed and non-exposed parts of the skin surface to the ambient air has still to be considered. Since in the present study the volunteers inhaled radon-free air (approximately 10 Bq m^−3^) through the breathing mask during the resting phase, inhalation of radon can also be neglected during this phase.

**Fig. 1:**
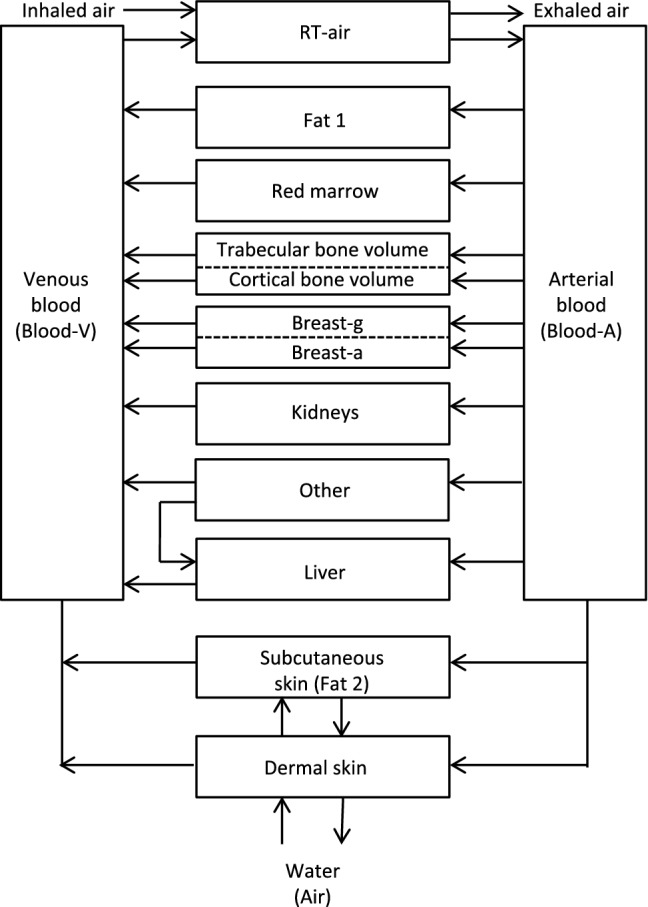
Structure of the biokinetic model used in the present study for the simulation of radon uptake through the skin in a thermal bath, based on the radon transfer and organ distribution model of Leggett et al. (2013), extended by two interacting skin compartments, the dermal skin (DS) and the subcutaneous skin (SS). The SS compartment replaces the fat 2 compartment of the Leggett et al. (2013) model. The DS compartment is subdivided into an exposed skin compartment (90%), i.e. exposed to thermal water, and a non-exposed skin compartment (10%), i.e. exposed to ambient air. *RT-air* respiratory tract air, *Breast-g* glandular tissue of breast, *Breast-a* adipose tissue of breast

The structure of the biokinetic radon transfer model for the simulation of radon uptake through the skin and subsequent exhalation used in the present study is illustrated in Fig. 1. The coupled linear differential equations describing the radon activity concentrations in the different organs and tissues as a function of time during and after the radon exposure were solved numerically with the program package Mathcad.

Finally, the experimentally observable quantity radon transfer (RT) proposed by Grunewald et al. (2009) to describe the total radon activity transferred from the water through the skin to the air exhaled from the lungs can be calculated by

6 $$ {\text{RT}} = {\text{RMV }}\int\limits_{{t_{1} }}^{{t_{2} }} {C_{\text{EX}} \left( t \right)} \;{\text{d}}t $$

where RMV is the average respiratory minute volume (i.e. the volume of air inhaled in 1 min) during exposure and resting, *C*_EX_(*t*) the radon activity concentration in the exhaled breath, and *t*_1_ and *t*_2_ the time at the onset of the exposure and at the end of the resting phase, respectively. In experimental terms, the integral is given by the area under the time-dependent exhalation function within *t*_1_ and *t*_2_.

## Results and discussion

### Results of exhalation measurements

**Fig. 2:**
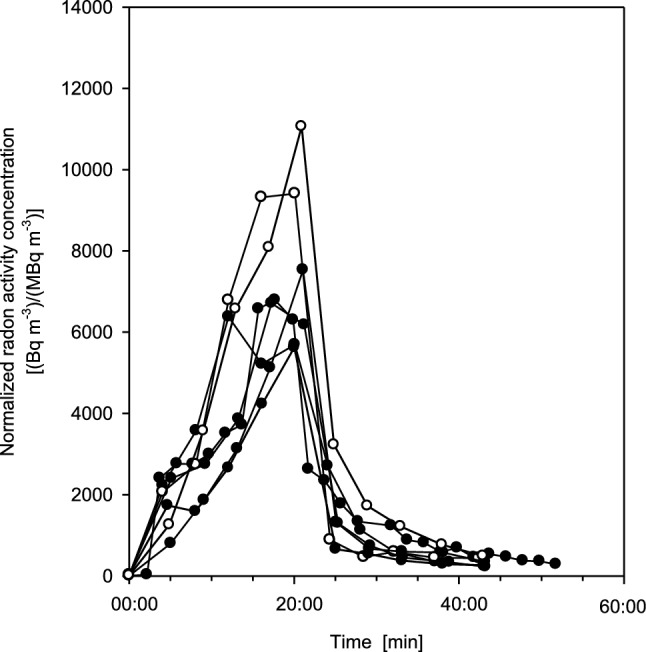
Individual exhalation curves for seven measurements (six test persons, one measured twice), normalized to the corresponding mean radon activity concentration in thermal water [Bq m^−3^/(MBq m^−3^)], for 20 min bathing and subsequent 20 min resting phases. The variations of the exhalation curves among the six volunteers illustrate the effect of inter-subject variability. Full circles denote female and open circles male volunteers. Estimated statistical errors of the individual measurements range from ± 5 to 15%

Individual exhalation curves as a function of time for seven measurements (six test persons, one measured twice) are plotted in Fig. 2, normalized to the corresponding radon activity concentrations in thermal water. Upon entering the bathtub, the radon activity concentration in the exhaled breath rapidly increased almost linearly with time, reaching a maximum value at the end of the exposure, and then decreased rapidly almost exponentially with time within several minutes during the subsequent resting phase. The apparent variations of the exhalation curves among the six volunteers illustrate the effect of inter-subject variations among the test persons. In general, however, the shapes of the exhalation curves are relatively similar, except for their maximum values, which are higher by nearly a factor of 2 for the two male subjects as compared to the four female volunteers. This gender difference may be caused primarily by the higher body surface areas of the male test persons (Table 2), as body surface area determines the uptake of radon through the skin.

**Fig. 3:**
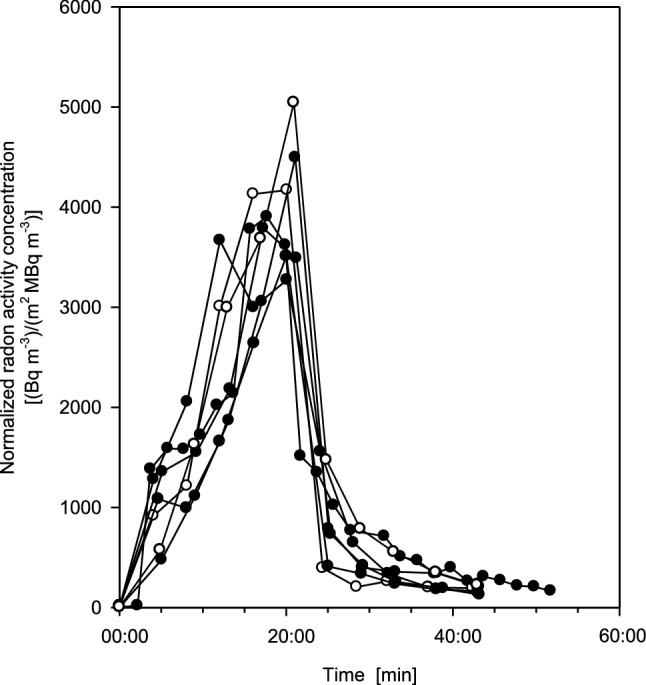
Individual exhalation curves for seven measurements (six test persons, one measured twice) normalized to both radon activity concentration in thermal water and individual body surface area [(Bq m^−3)^/(m^2^ MBq m^−3^)], for 20 min bathing and subsequent 20 min resting phases. The additional normalization greatly reduces inter-subject variations. Full circles denote female and open circles male volunteers. Estimated statistical errors of the individual measurements range from ± 5 to 15%

Therefore, in Fig. 3 the measured values plotted in Fig. 2 are further normalized to the individual body surface areas which indeed, as compared to Fig. 2, reduced the differences between the exhalation curves for female and male volunteers significantly. Any variations still observable may primarily represent inherent individual differences in breathing patterns, blood transport parameters among the organs, and skin permeability. Alternatively, exhalation curves were also normalized to individual body mass. Since body mass and surface area are correlated, similar results were obtained which are not presented here.

**Fig. 4:**
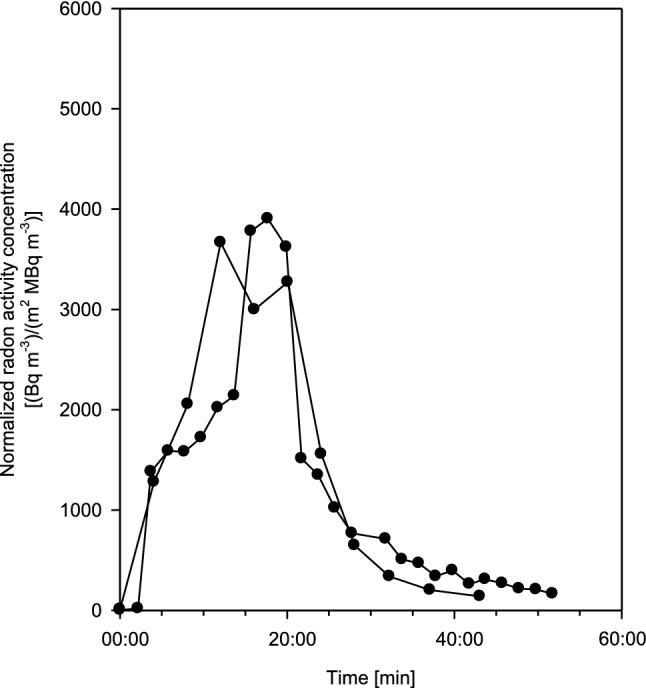
Two exhalation curves for test person 1 (female) at different times, normalized to both radon activity concentration in thermal water and individual body surface area, for 20 min bathing and subsequent 20 min resting phases, illustrating the effect of intra-subject variability. Estimated statistical errors of the individual measurements range from ± 5 to 15%

Because individual breathing patterns may vary between two measurements, volunteer 1 (female) was measured twice at different times. Resulting exhalation curves, normalized to both individual radon activity concentration in thermal water and body surface area, are plotted in Fig. 4, illustrating intra-subject variations. While the overall pattern of the exhalation curve is similar in both measurements, differences in the length of the plateau and the shape of the rising portion of the exhalation curve can be observed. Since the experimental setup was the same in both measurements, the most likely reason for this difference is the daily variability of the breathing parameters.

**Fig. 5:**
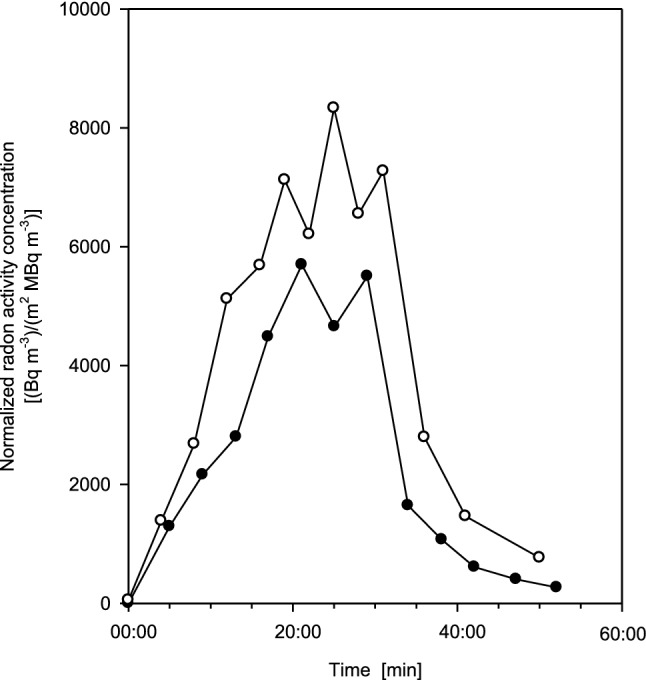
Exhalation curves for test person 4 (female) and 6 (male), normalized to both radon activity concentration in thermal water and individual body surface area for 30 min radon exposure and subsequent 20 min resting phase, demonstrating the effect of saturation. Full circles denote female and open circles male volunteers. Estimated statistical errors of the individual measurements range from ± 5 to 15%

Previous exploratory measurements with one test person (Hofmann and Winkler-Heil 2010) showed the measured exhalation curve to continuously approach a saturation value towards the end of exposure, followed by an exponential decrease with time. In the present study, however, the exhalation curves of only one-half of the participants exhibited this saturation pattern during the exposure phase, while those of the others reached a maximum value after 20 min without saturation. Note, however, that no differences could be observed for the resting phase. Thus, to test the possible development of a saturation level, the exposure period was prolonged to 30 min for two individuals. The resulting exhalation curves for test persons 4 (female) and 6 (male), normalized to individual radon activity concentration and body surface area are plotted in Fig. 5. Test person 4 already exhibited saturation after 20 min of exposure, and the additional 10 min simply extended the saturation phase. In contrast, test person 6 did not reach saturation after 20 min, but after 30 min. This indicates that the exhalation curves of all test persons will eventually exhibit saturation, although at different times of exposure.

**Fig. 6:**
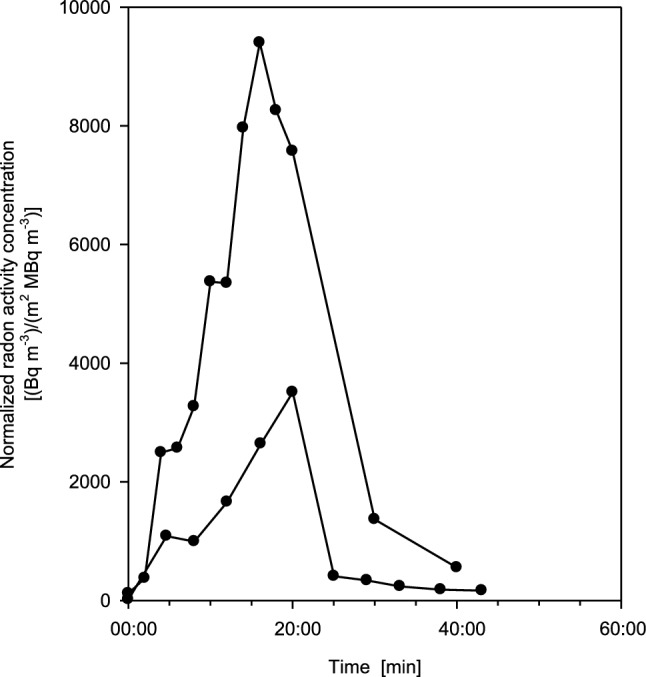
Comparison of exhalation curves for test person 2 (female), normalized to both radon activity concentration in thermal water and individual body surface area, between normal exposure (full circles) and pre-heating in radon-free water of the same temperature for 10 min (full squares), for both 20 min bathing and subsequent 20 min resting phases. This comparison demonstrates the effect of water temperature on the radon transfer through the skin and, in further consequence, the exhalation rate. Estimated statistical errors of the individual measurements range from ± 5 to 15%

While the blood flow rates assumed in the Leggett et al. (2013) model refer to environmental air temperatures, the blood flow through the external tissues of the body will probably be higher in thermal water at temperatures between 37 and 40 °C. In addition, the uptake of radon through the skin may also be affected by the water temperature. This temperature effect is illustrated in Fig. 6, where exhalation curves for test person 2 (female), normalized to radon activity concentration in thermal water and individual body surface area, are compared between normal exposure and prior pre-heating for 10 min in radon-free water of the same temperature. As shown in Fig. 6, the measurement with prior pre-heating in radon-free water showed a significant increase of the radon activity concentration in the exhaled air by a factor of about 2.5 over the whole exposure period, demonstrating that skin permeability and/or blood flow rate increase with temperature during the exposure phase.

### Results of model simulations

#### Adaptation of model parameters

As a result of the simulation of the radon transport in the human body using a system of linear differential equations, the shape of the exhaled radon activity concentration exhibits, by definition, a [1 − exp(− *λ*_E_*t*)] increase during the exposure phase and an exp(− *λ*_E_*t*) decrease during the resting phase (Hofmann and Winkler-Heil 2010; Sakoda et al. 2016). However, this theoretical behavior during the exposure phase differed from the actually measured exhalation profiles which showed an approximately linear increase with time since exposure without any appreciable saturation effect in some of the measurements. In contrast, the simulated radon exhalation curves during the resting phase were consistent with the measured data. This suggests that transfer rates of radon through the skin and its subsequent transport by the blood, originally derived for the uptake from air at normal temperatures (Leggett et al. 2013; Peterman and Perkins 1988), are modified for the transfer from water at higher temperatures during the exposure phase.

Two factors may be responsible for this temperature effect:

First, a higher peripheral body temperature widens the arterial blood vessels (the deeper lying venous blood vessels are less affected) and hence increases the flow of the arterial blood through the DS and SS compartments (Charkoudian 2003; Johnson et al. 1984; Rowell 1990; Taylor et al. 1984). For example, an increased body temperature of 40 °C increases the cardiac output roughly by a factor 2 and redistributes the blood flow from the core to the periphery (Rowell 1990). The resulting cutaneous vasodilation increases the blood flow to the skin several times, e.g. from about 0.5 l min^−1^ at 32 °C to about 8.5 l min^−1^ at 40 °C (Rowell 1990) which, however, can vary markedly among different individuals and even in the same individual (Johnson et al. 1984). The vasodilatory response to a warming stimulus is biphasic (Charkoudian 2003): there is an initial rapid increase in blood flow during the first 3–5 min, then a moderate decrease, and then a slow vasodilation that attains a plateau after 25–30 min.

Second, with increasing time in thermal water, the skin becomes more permeable as the warm water opens the pores. For example, Potts and Francoeur (1990) reported a four-fold increase in permeability of the skin for an increase in temperature from 22 to 40 °C. In addition, the diffusion coefficient increases with increasing temperature. Thus, the permeability coefficient increases with time in the thermal bath until eventually reaching a saturation value. Indeed, these modeling assumptions are supported experimentally by the pre-heating measurement illustrated in Fig. 6. The apparent indentation of the exhalation curve after about 10 min of exposure observed in some exhalation curves suggests that both effects saturate after about 10 min (Charkoudian 2003).

While the adapted model correctly predicts a saturation effect observed in about half of the measured exhalation curves, it does not capture the saturation at the end of exposure in the other curves. This saturation can only be achieved by assuming that the permeability coefficient starts to decrease again after about 10–15 min. A potential mechanism for this behavior is the swelling of the stratum corneum, the outermost layer of the skin. For example, Silva et al. (2007) reported a substantial swelling of corneocytes in the case of high water content of the stratum corneum. Indeed when immersed in water, the horny layer of the skin could swell vertically to 4–5 times of its original thickness, thereby reducing the diffusional transport through this layer (Kligman 2000) and, consequently, the permeability coefficient of the dermal skin.

To include the effects of both water temperature and skin swelling in the prediction of exhalation curves, the numerical values of the DS permeability coefficient, *K*, the transfer coefficient from DS to SS, *k*_DS_, and the arterial blood flow rates, *F*_SD_ and *F*_SS_, have to be modified. It is noted that the numerical value of the skin permeability coefficient in the Sakoda et al. (2016) model considers already the combined effect of water temperature and skin swelling. However, using this value leads to a rather steep increase of the exhalation curve together with a distinct saturation effect, which was not observed in the present study.

For modeling purposes, the effect of the water temperature on the DS permeability coefficient and the arterial blood flow rate were expressed by time-dependent linear functions obtained by fitting the exhalation curve of the pre-heating experiment. More specifically, the skin permeability coefficient used in the Sakoda et al. (2016) model was linearly increased with time up to a factor 4 from *t* = 0 to *t* = 10 min. Likewise, the blood flow rates to both skin compartments at normal temperatures were also linearly increased with time up to a factor 4 during the first 10 min. The resulting values at 10 min were subsequently adopted for the remaining 10 min of the exposure time.

To consider the effect of the water uptake on the skin permeability, the skin permeability coefficient was modified to match the exhalation curves exhibiting a saturation effect. Thus, the value of *K* was reduced linearly with time by a factor 2.5, from the value at *t* = 12.5 min to the end of the exposure. Although the swelling effect of the skin may have already started at an earlier stage, its decreasing effect may have been overruled by the increasing effect of the water temperature. Indeed, the absence of an observable saturation effect in about half of the measured exhalation curves suggests that the effect of swelling of the skin is small compared to the effect of the water temperature on skin permeability and arterial blood flow, in these individuals.

While the temperature effect dominates the permeability during the first 10 min, swelling will be effective primarily during the last 15–20 min in the bathtub. Conversely, the lower water content during the resting phase, together with the negative temperature effect, will again increase the permeability of the skin.

Furthermore, it was assumed that water temperature and skin swelling affects not only the transfer from water to dermal skin but also the transfer coefficient from DS to SS, *k*_DS_. Thus, for modeling purposes the dependence of *K* on water temperature and skin swelling was also adopted for *k*_DS_, although assuming a weaker dependence, i.e. about 20%.

Because the measured exhalation curves reflect the combined effect of skin permeability and blood flow, they do not allow to quantify both effects separately. Fortunately, computed exhalation curves were only moderately sensitive to parameter variations and, thus, different combinations of these parameters produced similar exhalation curves, within the statistical errors of the measured values. Moreover, these simulations indicated that the influence of the arterial blood flow rates is small compared to that of the permeability coefficient.

#### Comparison with measured exhalation curves

**Fig. 7:**
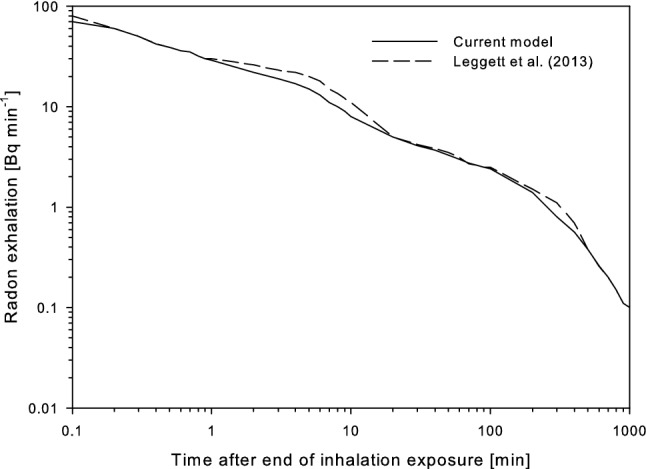
Comparison of model predictions of the exhalation rate of radon following continuous exposure to a radon activity concentration of 25.9 Bq l^−1^ for 8.5 h with the model simulations of Leggett et al. (2013)

To validate the current model, the radon exhalation rate was calculated for the exposure to a constant elevated radon activity concentration in air for 8.5 h and a subsequent wash-out over a period of about 120 h (Harley et al. 1994), and the results were compared to the model predictions of Leggett et al. (2013), revealing excellent agreement (Fig. 7). This suggests that the structural and parametric modifications of the Leggett et al. (2013) model performed in the present study did not affect the overall behavior of the original model.

**Fig. 8:**
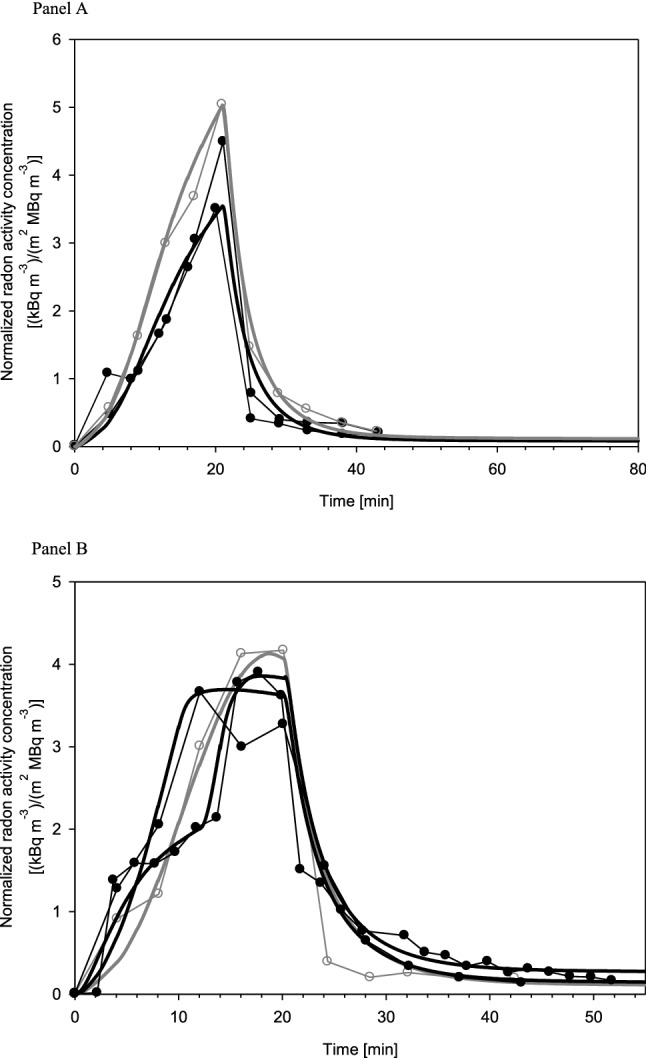
Measured exhalation curves normalized to the same Rn activity concentration in water and individual body surface area: comparison of measured values with model predictions for 20 min bathing and subsequent 20 min resting phases. Simulations were based either on exhalation curves with no saturation (**a**) or on exhalation curves with an observable saturation (**b**). Full circles denote female and open circles male volunteers

Furthermore, the radon exhalation curves calculated with the present model for an exposure of 20 min are compared in Fig. 8 to the exhalation curves measured and normalized to the same radon activity concentration in water and individual body surface area. Since Fig. 3 suggests that three measured exhalation curves exhibited a saturation behavior at the end of the 20 min exposure phase, while the four other measured exhalation curves (note: one test person was measured twice) showed no saturation, simulations were carried out separately for both groups.

Simulations for the three subjects without saturation are compared in Fig. 8a with the corresponding experimental data. For these simulations, both the permeability coefficient and the transfer coefficient from dermal to subcutaneous skin were expressed as functions of the water temperature. The simulations revealed that the permeability coefficient affects both the initial slope of the exhalation curve as well as its maximum value at the end of the exposure, i.e. the higher the permeability coefficient, the steeper is the slope and the higher is the maximum value. While all simulations were based on the same set of parameter values, only the absolute value of the permeability coefficient, *K*, was varied to reproduce the measured exhalation curves.

Corresponding simulated exhalation curves with saturation are shown in Fig. 8b. Here the situation is more complex because three different shapes can be observed: (1) a relatively continuous increase with time followed by saturation, (2) a steeper increase with a prolonged saturation level, and, (3) a change in the initial slope after about 5 min and then again after about 10 min (this change after about 5 min has also been observed in other exhalation curves). Note that saturation requires not only both the permeability coefficient and the dermal-subcutaneous skin transfer coefficient to depend on temperature, but also to depend on the water content of the skin in the second half of the exposure. Thus, the three exhalation curves were simulated by different combinations of the permeability coefficient and the dermal-subcutaneous skin transfer coefficient as a function of temperature and skin water content.

The individual respiratory parameters and body characteristics of the six volunteers participating in this study (Table 2) which affect the uptake of radon through the skin and the exhalation of radon from the lungs are the skin surface area *A*_DS_ (*A*_DS_ = 0.9 BSA) (see Eq. 4), the respiratory tract volume *V*_RT_, and the transfer coefficient from the respiratory tract air to the environment *λ*_E_, the latter depending on the breathing rate, RMV (see Eqs. 2 and 3). While the present simulations are based on values recommended by ICRP (1994, 2002), these values may differ from the specific values of the volunteers. Therefore, to investigate the influence of individual body surface area, respiratory tract volume and minute volume on the radon exhalation curves, different combinations of parameter values were used in the simulations.

**Fig. 9:**
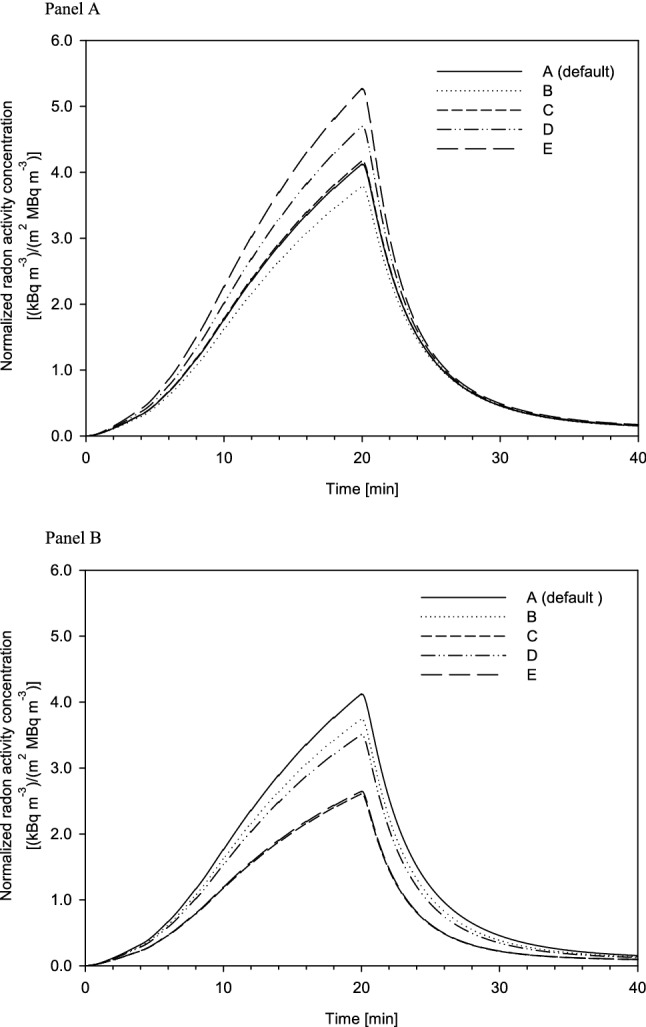
Inter-subject variability due to individual anatomical (**a**) and respiratory parameters (**b**) of the volunteers, affecting the uptake of radon through the skin and the exhalation of radon from the respiratory tract, respectively. **A** Variation of body surface area (BSA): (A) default value (females) = 1.61 m^2^, (B) BSA (females) = 1.52 m^2^ (min), (C) BSA (females) = 1.68 m^2^ (max), (D) BSA (males) = 1.90 m^2^ (ICRP 2002), and (E) average individual BSA (males) = 2.14 m^2^. **b** Variation of respiratory tract volume (*V*_RT_) and respiratory minute volume (RMV): (A) default values (females) = 3.13 l and 390 l h^−1^, (B) average individual *V*_RT_ = 3.10 l and RMV = 434 l h^−1^ (min) (females), (C) *V*_RT_ = 3.10 l and RMV = 640 l h^−1^ (max) (females), (D) average individual *V*_RT_ = 4.17 l and RMV = 466 l h^−1^ (min) (males), and (E) *V*_RT_ = 4.17 l and RMV = 640 l h^−1^ (max) (females)

While the total surface areas of the human body for adults recommended by ICRP (2002) are 1.90 m^2^ for adult males and 1.66 m^2^ for adult females, the corresponding average values for the male and female volunteers in the present study were 2.14 m^2^ and 1.61 m^2^, respectively. Thus, the surface area of the male subjects was significantly higher than the ICRP (2002) value, and their individual values were very similar. In contrast, the surface area values for the female subjects ranged from 1.52 to 1.68 m^2^, and their average surface area was quite similar to the ICRP (2002) value. The effect of body surface area on the exhaled radon activity concentration is illustrated in Fig. 9a for exhalation curves without saturation. Since the simulations shown in Fig. 8 were consistently based on the female ICRP value of 1.61 m^2^, this value serves as the control scenario in this figure (default value). Compared to the control scenario, most subjects show higher radon activity concentrations in the exhaled air. As expected, the maximum values of the exhalation curves are proportional to body surface area. This demonstrates that individual body surface area is a major contributor to the inter-subject variability of the measured radon exhalation curves.

Compared to the volumes of the respiratory tract air for adult males and females recommended by ICRP (1994), i.e. 3.86 l and 3.13 l, respectively, average RT volumes derived for the individual subjects were 4.17 l and 3.10 l, respectively. Individual respiratory tract volumes were derived as the sum of the individual FRC and the extrathoracic volume V_ET_, the latter scaled in proportion to the FRC (ICRP 1994). Note that RT volumes within each gender group were quite similar (see Table 2). While the average volume for the female subjects is very similar to the ICRP (1994) value, the average value for the male subject is about 10% higher.

Furthermore, the transfer coefficient from RT-air to the environment, *λ*_E_, depends on the breathing rate. Thus, to consider the individual breathing patterns of the test persons, the default value of 2600 d^−1^ (Leggett et al. 2013), which approximates sitting awake breathing conditions (ICRP 1994), was replaced by the measured respiratory parameters, scaling *λ*_E_ by the ratio of the individual RMVs of the test persons to the corresponding ICRP (1994) values for adult males and females. While the average RMV of the male volunteers of 536 l h^−1^ is virtually identical to the ICRP value of 540 l h^−1^, the average RMV of the female volunteers of 514 l h^−1^ is considerably higher than the ICRP value of 390 l h^−1^, probably as a result of the somewhat artificial breathing. Moreover, significant variations within each gender group can be observed (Table 2). Compared to the control scenario (default value) plotted in Fig. 9 (*V*_RT_ = 3.13 l, RMV = 390 l h^−1^) (ICRP 1994, 2002), all combinations of *V*_RT_ and RMV values produce lower radon activity concentrations in the exhaled air. Since higher respiratory tract air volume and respiratory minute volume both decrease the radon activity concentration in the respiratory tract air, higher *V*_RT_ and RMV values in the male subjects reduce the exhaled radon activity concentration as compared to females. This suggests that the higher body surface area of the two male volunteers as compared to the females, which increases radon uptake through the skin, and the higher respiratory tract air volume and minute volume, which decreases the radon activity concentration in the respiratory tract air, somewhat balance each other. Corresponding simulations for the exhalation curves with saturation revealed similar results and thus are not discussed here.

Although the fat content in subcutaneous skin is not explicitly considered in Eqs. 4 and 5, it may affect the transfer coefficient from dermal to subcutaneous skin, *k*_DS_, and hence the uptake of radon from thermal water. Since fat 2 (or subcutaneous fat) in both Leggett et al. (2013) and Peterman and Perkins (1988) models represents 50% of the total body fat, subcutaneous skin fat in the volunteers ranged from 5.4 to 10.5 kg (see BF values in Table 2). While the average value of 7.7 kg of the female volunteers is similar to the ICRP (2002) average value of 8.0, the average value of 10.2 kg for the male volunteers is much higher than the corresponding ICRP (2002) value of 6.8 kg. Unfortunately, no information on the relationship between the subcutaneous fat content and the transfer coefficient *k*_DS_ is currently available. This is unfortunate because exploratory simulations performed in the present study indicated that individual variations of the fat content will significantly contribute to the observed inter-subject variability of exhalation curves.

For comparison, the application of gender-specific values for transfer coefficients, blood flow rates and compartment volumes provided by Leggett et al. (2013) for females and males only slightly affected the radon exhalation curves. Therefore, the observed slight variations can be neglected compared to the variations due to inter-subject differences of individual body surface area, respiratory air volume and respiratory minute volume.

#### Distribution among organs and tissues

**Fig. 10:**
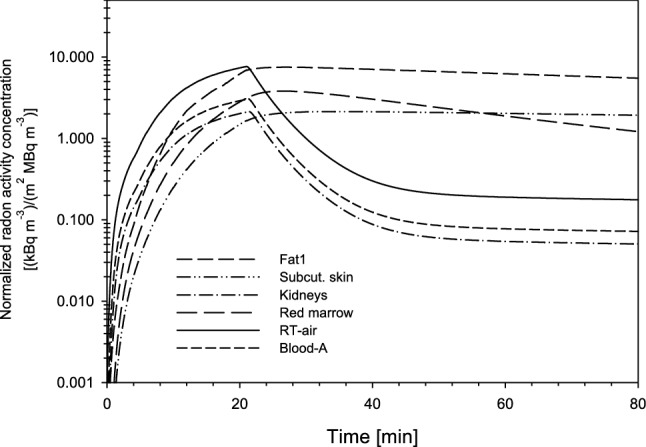
Radon activity concentrations in selected organs and tissues of the human body, normalized to both radon activity concentration in thermal water and individual body surface area, as a function of time during 20 min radon exposure in the thermal bath and subsequent 20 min resting phase, based on exhalation curves with no saturation (shown in Fig. 8a)

Based on the parameters of the Leggett et al. (2013) and Sakoda et al. (2016) models and the additional model parameters derived in the present study by comparison of the simulated with the experimental exhalation curves, radon activity concentrations in several selected organs of the human body (i.e. respiratory tract air, arterial blood, fat 1, subcutaneous skin (fat 2), kidneys and red marrow) were simulated as a function of time during and after radon exposure in the thermal bath. The results are shown in Fig. 10 for the measurements without saturation (see panel A of Fig. 8). Two different patterns can be distinguished:

The radon activity concentrations in the air of the respiratory tract, the arterial blood, and the kidneys increase during the exposure phase and then drop off immediately after the end of exposure, in line with the behavior of the radon exhalation curves. The radon activity concentrations in venous blood, which supports the respiratory air compartment, are practically indistinguishable from those in the respiratory air. In contrast, the radon activity concentrations in red marrow, fat 1 and subcutaneous skin (fat 2) also increase during exposure, but then remain nearly constant for some time during the resting phase, and subsequently decrease at a relatively slow rate. This slow decrease in radon activity concentration reflects the storage capacity of the fat in these compartments. Consistent with the radon exhalation curves, the permeability coefficient does not affect much the maximum values of the radon activity concentrations in the different organs, nor does it affect their initial slopes. Since all other compartments are supported by the arterial blood, the radon activity concentration in these compartments shows a similar behavior as that in arterial blood, although absolute values differ as a result of differences in blood flow rates.

Simulations of the three measured exhalation curves with saturation revealed that the radon concentrations curves for RT-air, venous blood and kidneys follow closely the corresponding radon exhalation curves during the exposure phase. However, differences among the three exhalation curves during the exposure phase (Fig. 8b) do not affect the shapes of the radon activity concentration curves in those organs, which do not immediately decline after the end of the exposure. Furthermore, the absolute values of the radon activity concentrations in all organs in the subsequent resting phase are determined by their maximum activity concentrations in exhaled air at the end of the exposure, which are different between the three exhalation curves.

**Fig. 11:**
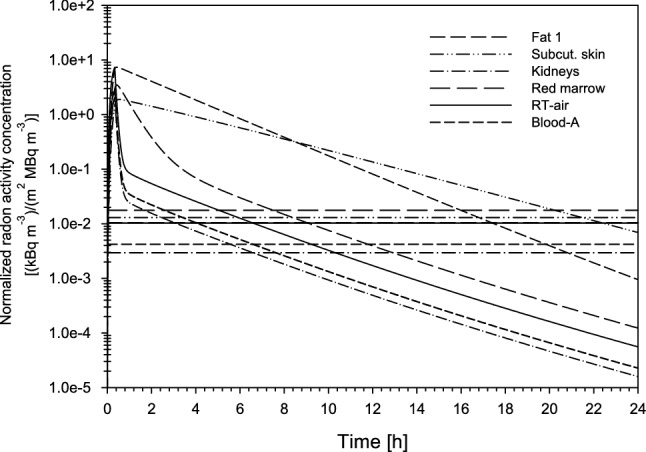
Radon activity concentrations in selected organs and tissues of the human body, normalized to both radon activity concentration in thermal water and individual body surface area, as a function of time during 20 min radon exposure in the thermal bath and a subsequent period of 24 h, based on exhalation measurements with no saturation. The corresponding radon activity concentrations resulting from a continuous inhalation of 100 Bq m^−3^ represent the contribution from environmental exposure to radon

As a result of the relatively slow decline of the radon activity concentrations in some compartments, the corresponding organs and tissues will still receive radiation doses for some time after the end of the exposure. This is illustrated in Fig. 11 where the reduction of the radon activity concentrations simulated for several selected organs of the human body during the following 24 h is shown. To determine the exclusive effect of radon uptake through the skin, the radon activity concentration in the inhaled air was set equal to zero in these simulations. While the radon activity concentrations in those organs or tissues with a quick decline immediately after the end of exposure drop to lower levels within the first few hours, fat 1 and subcutaneous skin (fat 2) exhibit a slower decline and, consequently, experience a prolonged exposure to alpha particle irradiation.

After the end of the resting period and outside the therapy facilities, patients are in addition continuously exposed to environmental radon levels. The resulting continuous radon inhalation further contributes to the radon activity concentrations in the different organs and tissues of the patients, producing constant activity concentration levels. To illustrate the unavoidable contribution of environmental radon, radon activity concentrations in the selected organs and tissues resulting from the inhalation of 100 Bq m^−3^ (average indoor radon level in the Gastein area) are included for comparison. It turns out that after a few hours, the radon activity concentrations in most organs are solely produced by the inhalation of environmental radon, except for the two fat compartments where the effect of radon uptake in the thermal bath still persists up to about 24 h.

#### Radon transfer

The quantity “radon transfer” (RT) proposed by Grunewald et al. (2009) to describe the total radon activity transferred from the water through the skin to the air exhaled from the lungs can be calculated by determining the area under the measured exhalation curve between start and end of exposure multiplied by the exhaled air volume. Individual total RT values for a 20 min radon exposure are compiled in Table 3, ranging from 655 to 1176 Bq, with an average value of 814 Bq. Normalization to individual radon activity concentrations in water and body surface areas reduces the range of the radon transfer values somewhat, e.g. from 394 to 609 Bq/(MBq m^−5^), with an average value of 522 Bq/(MBq m^−5^). While RT values for 30 min exposures were higher by about a factor of 2, pre-heating also increased the radon transfer.

**Table 3:** Individual radon transfer in radon thermal water therapy for a bathing time of 20 min, based on the measured exhalation data (RT), normalized to the corresponding radon activity concentrations in water (RT/Rn), and additionally normalized to individual body surface area (RT/(Rn BSA)

Subject	Radon transfer RT (Bq)	Normalized radon transfer	
		RT/Rn	RT/(Rn BSA)
		[Bq/(MBq m^−3^)]	[Bq/(MBq m^−5^)]
1	655	899	545
	851	939	569
2	658	778	512
	1718^a^	1909^a^	1256^a^
3	612	626	394
4	689	771	459
	1479^a^	1732^a^	1031^a^
5	1176	1304	609
6	1055	1182	568
	2728^a^	3213^a^	1545^a^
	1559^b^	2267^b^	1090^b^

^a^30 min bathing time

^b^Pre-heating in radon-free water at *T* = 37 °C for 10 min, followed by 20 min bathing

For comparison, Grunewald et al. (1999) reported an average total RT value of 1000 Bq for 20 min exposures at 2 MBq m^−3^, which corresponds to about 450 Bq if converted to a radon activity concentration of 0.9 MBq m^−3^. A similar value of 460 could be derived from the measurements of Grunewald et al. (2009) for a 40 min exposure at 1.5 MBq m^−3^. The higher RT values obtained in the present study, 814 versus 450, is caused by the steep increase of the exhaled radon activity concentrations right at the onset of exposure, while the exhalation curves in the Grunewald et al. (1999, 2009) measurements increased much slower in a sigmoid fashion.

## Conclusions

The apparent variations of the measured exhalation curves among the six volunteers, consisting of four female and two male subjects, normalized to the corresponding radon activity concentration in thermal water (Fig. 2), illustrate the effects of female-male differences and inter-subject variations among the test persons. This inter-subject variability of the exhaled radon activity concentrations is caused by variations of individual body characteristics and respiratory parameters, such as body surface area, the content of body fat, respiratory tract volume, and respiratory minute volume. While the effect of these individual parameters could be quantified for each test person (Table 2), inter-subject variations of the transfer rates, blood flow rates and organ or tissue volumes of the Leggett et al. (2013) model may also have played a role but could not be estimated individually. The results of the present study suggest that the variability of the permeability coefficient of the skin, the body surface area and the fat content of the subcutaneous skin may be the most important individual parameters affecting the uptake of radon from thermal water. In addition, the individual dependence of skin permeability and blood flow rates on water temperature as well as the degree of skin swelling may also contribute to the observed variability in radon uptake among different persons.

Although the basic shapes of the measured exhalation curves were relatively similar, the slopes of the increasing part of the exhalation curves and their maximum values attained at the end of exposure were quite variable. Most notably, the exhalation curves of about one-half of the participants showed some saturation towards the end of exposure, while the others exhibited no saturation effect. Compared to the exposure phase, differences between the measured exhalation curves during the resting phase were relatively small. As a consequence of these inter-subject variations, the experimentally observed individual exhalation curves could not be simulated by the same set of parameter values but required the use of individual person-specific values.

In a previous preliminary study of some of the authors of the present paper (Hofmann and Winkler-Heil 2010) and in the recent study of Sakoda et al. (2016), where radon uptake, transfer and exhalation was modelled by a system of linear differential equations, the resulting radon exhalation curves were relatively steep during the first part of the exposure phase followed by a distinct saturation at the end of exposure. However, this shape of the simulated exhalation curves was not compatible with the exhalation curves measured in the present study, exhibiting a much slower increase without a less pronounced, or even missing saturation level. In the present study, two novel features were included in the model to improve the agreement of the model predictions with the experimental evidence: (1) the inclusion of a subcutaneous fat compartment between skin and venous blood reduced the steepness of the exhalation curves and, (2) the skin permeability coefficient and the arterial blood flow were assumed to depend on water temperature which also reduced the initial slope, while the newly introduced process of skin swelling determined the saturation level. The shapes of the exhalation curves indicated that the temperature effect dominates the permeability during the first 5–10 min, while the skin swelling is effective primarily during the last 10–20 min.

While the radon activity concentrations in all organs increased during the exposure phase, although with different slopes, the related activity concentration curves during the resting phase either decreased rapidly, or they remained at a constant level or decreased slightly. These differences in uptake and removal rates are caused by organ-specific differences of transfer rates, blood flow rates and partition coefficients. It was observed that organs or tissues with prolonged radon activity concentrations still received radiation doses for up to 1 day after the end of the treatment session. The integral of the radon activity concentration in each organ from the onset of exposure to its complete elimination determines the total amount of radiation energy emitted by the radioactive decay of radon, which may then be related to the therapeutic response. Furthermore, the observed inter-subject variations of the radon uptake through the skin led to corresponding inter-subject variations of the radon activity concentrations and resulting radiation doses in the different organs and tissues. In addition, prolonged radon activity concentrations in a given organ may give rise to the formation of short-lived radon progeny, which may also contribute to the absorbed dose incurred in that organ.

If one intends to use the findings obtained in the present study for therapeutic treatment of patients, two aspects must be considered: (1) the experimentally observed inter-subject variability of the transport of radon through the skin, which leads to corresponding variations of the radon activity concentrations in different organs and tissues, must be considered for each patient individually, i.e. no uniform therapeutic response can be expected; and, (2) while the results presented in this study are based on experiments with young and healthy adults, the inter-subject variations may be even higher in older patients with a variety of diseases.

A previous study demonstrated that the deposition of radon progeny on skin surfaces may play a major role in the therapeutic response after radon exposure in a thermal bath (Tempfer et al. 2010). Indeed, the present model prediction that subcutaneous fat receives the highest radiation doses among all organs and tissues further emphasizes the role of the skin in radon balneotherapy. It is concluded that both radon transfer through the skin and attachment of radon progeny to the skin may contribute to the therapeutic effect of radon thermal baths.
